# Human Amnion-Derived Mesenchymal Stromal/Stem Cells Pre-Conditioning Inhibits Inflammation and Apoptosis of Immune and Parenchymal Cells in an In Vitro Model of Liver Ischemia/Reperfusion

**DOI:** 10.3390/cells11040709

**Published:** 2022-02-17

**Authors:** Giovanni Zito, Vitale Miceli, Claudia Carcione, Rosalia Busà, Matteo Bulati, Alessia Gallo, Gioacchin Iannolo, Duilio Pagano, Pier Giulio Conaldi

**Affiliations:** 1 Research Department, IRCSS ISMETT (Instituto Mediterraneo per i Trapianti e Terapie ad Alta Specializzazione), 90127 Palermo, Italy; vmiceli@ismett.edu (V.M.); rbusa@ismett.edu (R.B.); mbulati@ismett.edu (M.B.); agallo@ismett.edu (A.G.); giannolo@ismett.edu (G.I.); dpagano@ismett.edu (D.P.); pgconaldi@ismett.edu (P.G.C.); 2 Ri.MED Foundation, 90127 Palermo, Italy; ccarcione@fondazionerimed.com

**Keywords:** liver transplant, ischemia/reperfusion, inflammation, apoptosis, hAMSCs, priming, 3D culture

## Abstract

Ischemia/reperfusion injury (IRI) represents one of the leading causes of primary non-function acute liver transplantation failure. IRI, generated by an interruption of organ blood flow and the subsequent restoration upon transplant, i.e., reperfusion, generates the activation of an inflammatory cascade from the resident Kupffer cells, leading first to neutrophils recruitment and second to apoptosis of the parenchyma. Recently, human mesenchymal stromal/stem cells (hMSCs) and derivatives have been implemented for reducing the damage induced by IRI. Interestingly, sparse data in the literature have described the use of human amnion-derived MSCs (hAMSCs) and, more importantly, no evidence regarding hMSCs priming on liver IRI have been described yet. Thus, our study focused on the definition of an in vitro model of liver IRI to test the effect of primed hAMSCs to reduce IRI damage on immune and hepatic cells. We found that the IFNγ pre-treatment and 3D culture of hAMSCs strongly reduced inflammation induced by M1-differentiated macrophages. Furthermore, primed hAMSCs significantly inhibited parenchymal apoptosis at early timepoints of reperfusion by blocking the activation of caspase 3/7. All together, these data demonstrate that hAMSCs priming significantly overcomes IRI effects in vitro by engaging the possibility of defining the molecular pathways involved in this process.

## 1. Introduction

It is widely ascertained that liver disease is a growing cause of mortality worldwide [1]. Unfortunately, liver transplantation is, up to now, the only therapy for end-stage liver disease; thus, all the risks derived from this approach must be considered. Among others, ischemia/reperfusion injury (IRI) represents one of the leading causes of primary non-function acute liver transplantation failure [2]. IRI is a multistep damage due to inevitable ischemia of the organ upon resection, followed by reoxygenation of the organ (reperfusion). During the ischemic phase, which can vary according to the donor and to the quality of the organ, liver resections are normally subjected to preservation and interruption of the organ blood flow, leading to mitochondrial disfunction, ATP depletion, intracellular Ca^2+^ accumulation and a switch to the anaerobic metabolism [3]. During reperfusion, the restoration of blood supplies generates the activation of an inflammatory cascade along with ROS production, leading to neutrophil recruitment and apoptosis of endothelial and hepatic cells [4,5]. In particular, during liver IRI, Kupffer cells, resident liver macrophages [6], lose their tolerogenic anti-inflammatory phenotype, activating a pro-inflammatory cascade with the secretion of cytokines, including IL-1β, TNFα, IL-6 and IL-12 [7,8]. These molecules stimulate downstream the recruitment of neutrophils into the liver parenchyma, leading to further hepatic tissue damage [9] and the subsequent transplantation failure. Thus, the identification of new approaches to attenuate IRI effects becomes essential to improve the rate of successful organ transplant. In the past few decades, mesenchymal stromal/stem cells (hMSCs) and their cellular products, given their immunomodulatory, angiogenic and regenerative properties, have been considered important therapeutic tools for regenerative medicine and tissue repair [10,11,12,13,14]. In particular, hMSCs have been demonstrated to have anti-inflammatory properties that are crucial for hMSCs-based therapy for immune-mediated disease [15]. Therefore, in the last few years, different studies have been performed focusing on the protective effects of hMSCs and their cellular products for the treatment of ischemic diseases, including IRI [16,17,18,19]. In the majority of these studies, the MSCs’ source is represented by bone marrow and adipose tissues. However, our group recently demonstrated for the first time that human amnion-derived mesenchymal stromal/stem cells (hAMSCs) attenuated the effects of IRI in an in vitro model of human alveolar epithelial cells [20]. Interestingly, we found that the conditioned medium (CM) from hAMSCs cultured in 3D conditions further reverted the IRI phenotype. Thus, these findings led to the possibility that the priming conditions could increase the hMSC therapeutic potential for liver IRI damage. In this study, we set up an in vitro model of liver IRI, with particular interests in the molecular changes mediated by the injury on M1-like macrophages and hepatic cells. Furthermore, we evaluated the effects of 3D and IFNγ hAMSCs priming on reperfused immune and hepatic cells in order to assess the potential reversion of the IRI phenotype.

## 2. Materials and Methods

### 2.1. Cell Culture

Human epithelial hepatocyte cell line THLE-2 (ATCC, CRL-2706) was cultured at 37 °C in a humidified environment containing 5% CO_2_. THLE-2 culture was performed in bronchial epithelial cell basal medium with additives (BEGM from Lonza/Clonetics Corporation, Bend, OR, USA, BEGM Bullet Kit) and supplemented with 10% fetal bovine serum (FBS, Hyclone, Logan, UT, USA), 100 U/mL penicillin, 100 μg/mL streptomycin (Gibco Invitrogen, Waltham, MA, USA) and 70 ng/mL phosphoethanolamine (Sigma Aldrich, St. Louis, MO, USA).

### 2.2. Isolation of Monocytes and Differentiation in M1-Like Macrophages

Human monocytes were obtained from 6 healthy volunteers. Human peripheral blood mononuclear cells (PBMC) were isolated from venous blood by density gradient centrifugation on Lympholyte Cell Separation Media (Cedarlane Laboratories Limited, Burlington, ON, Canada). CD14^+^ monocytes were separated from PBMCs by immunomagnetic sorting using anti-CD14 (MACS CD14 Microbeads; Miltenyi Biotec, Auburn, CA, USA) magnetic microbeads [21]. Immunomagneting sorting efficiency was 98% according to flow cytometry analysis (data not shown). CD14^+^ monocytes were immediately subjected to macrophage differentiation, as described by Tarique and co-workers [22]. Briefly, CD14^+^ monocytes were cultured in RPMI 1640 medium (Sigma-Aldrich, USA) supplemented with 10% FBS, 1% penicillin/streptomycin (Sigma-Aldrich, USA), 10 mM HEPES (Euroclone, Pero MI, Italy) and 1 mM L-glutamine (Lonza, USA). M1-like Macrophage differentiation was performed with 50 μg/mL GM-CSF (Miltenyi Biotec, Auburn, CA, USA) for 5 days, followed by 4 days of 20 μg/mL LPS + 20 μg/mL IFNγ (Miltenyi Biotec, Auburn, CA, USA) treatment. M0-like macrophages were obtained after 50 μg/mL GM-CSF treatment for 9 days, while M2-like macrophages were differentiated with 50 μg/mL GM-CSF (Miltenyi Biotec, Auburn, CA, USA) for 5 days, followed by 20 μg/mL IL-4 + 20 μg/mL IL-13 (Miltenyi Biotec, Auburn, CA, USA) treatment for 4 days.

### 2.3. Flow Cytometry

M1-like macrophages were harvested, washed with 1X PBS and then stained with the following anti-human monoclonal antibodies: CD209-FITC, CD64-PE-Vio770, CD80-APC, CD86-PE (all from Miltenyi Biotec, Auburn, CA, USA). For each antibody, isotype controls were used according to fluorescence and antibody specificity (REA293 Isotype control antibody, human IgG1—APC, FITC and PE-Vio770, Miltenyi Biotec, Auburn, CA, USA). Cells were incubated for 20 min at RT in the dark. Cells acquisition has been performed with a 16-colors BD FACS Celesta SORP Cell Analyzer (BD Biosciences, San Jose, CA, USA) with the same instrument setting. At least 10^4^ cells were analyzed using Kaluza Version 2.1.1 software (Beckman Coulter, Carlsbad, CA, USA).

### 2.4. Isolation of Mesenchymal Stromal/Stem Cells from Human Amniotic Membrane

MSCs were isolated from placenta of healthy donors within 6 h of birth. Written informed consent and details of the procedure were approved by IRCSS-ISMETT’s Institutional Research Review Board (IRRB). To obtain the cells, the amnion membrane was cut into small pieces and each fragment was decontaminated, under sterile conditions at room temperature, in three different solutions: (1) PBS supplemented with 2.5% Esojod for 2–3 s (Esoform, Rovigo, Italy); (2) PBS supplemented with 500 U/mL penicillin, 500 mg/mL streptomycin, 12.5 mg/mL amphotericin B and 1.87 mg/mL cefamezin for 3 min (Pfizer, Milan, Italy); (3) PBS supplemented with 100 U/mL penicillin and 100 mg/mL streptomycin for 5 min. Decontaminated fragments were digested for 9 min at 37 °C in HBSS (Lonza, USA) containing 2.5 U/mL dispase (Corning, Corning, NY, USA), and then maintained for 5 min at room temperature in RPMI 1640 supplemented with 10% FBS. Afterward, the amniotic fragments were digested with 0.94 mg/mL collagenase A (Roche, Mannheim, Germany) and 20 mg/mL DNase (Roche, Mannheim, Germany) for 2.5 h at 37 °C. The cell suspension obtained was filtered with both 100 μm and 70 μm cell strainers (BD Falcon, Franklin Lakes, NJ, USA), pelleted and resuspended in RPMI 1640 medium supplemented with 10% FBS for cell counting. Isolated cells were cultured, until passages 3–5, in polystyrene culture dishes (Corning, USA) at 37 °C, 5% CO_2_ in Chang Medium (Irvine Scientific, Santa Ana, CA, USA), at a density of 1 × 10^4^/cm^2^. The hAMSCs were phenotypically characterized as previously described [20].

### 2.5. Preparation of Mesenchymal Stromal/Stem Cell Spheroids

The hAMSCs at the second passage were cultured in DMEM serum-free medium at 5% CO_2_ and 37 °C (5 × 10^5^ cells/mL). The cells were maintained in 2 mL of culture medium in a suspended state to allow the formation of three-dimensional spheroids in 6-well ultralow attachment plate (Corning, Corning, NY, USA) that facilitates spheroid formations and their maintenance.

### 2.6. Conditioned Media Preparation

For CM collection from 2D cultures, the cells at the second passage were plated (5 × 10^5^ cells/mL) in a 10 cm-dish (Nunc, Bremen, Germany) containing DMEM supplemented with 10% FBS until 90–95% confluence. The medium was then replaced with a serum-free DMEM with or without 200 IU/mL of IFNγ (Human IFN-g1b premium grade, Miltenyi Biotec, USA), and the cells were grown for 2 days to obtain both 2D hAMSC-CM and γ-hAMSC-CM. For CM collection from 3D cultures (3D hAMSC-CM), we first observed the initial spheroid formation for 1 day. Then, the medium was changed (by means of a gentle centrifugation), conditioned for 2 days and finally collected. At the end of cultures, each ml of collected medium was conditioned by 10^6^ cells. The supernatant from cultures was centrifuged to remove cell debris and frozen at −80 °C until use.

### 2.7. In Vitro Protocol of Ischemia/Reperfusion

M1-like macrophages and THLE-2 cells were subjected to cold ischemia and warm reperfusion in order to obtain the desired cell injury. Cold ischemia was performed at 4 °C at 5% O_2_ exposition for 5 h (M1-like macrophages) and 12 h (THLE-2 cells) in Hypothermosol preservation medium (Sigma Aldrich, USA). At the end of the ischemia induction, the cells were briefly re-warmed at room temperature for 30 min, then subjected to medium change (normal culture medium according to the cell type) for the warm static reperfusion that was performed at 5% CO_2_ and 37 °C. Reperfused cells were harvested for further analysis at 1, 4 and 24 h of reperfusion. For all the experiments in this manuscript, M1-like macrophages and THLE-2 were seeded at 4 × 10^4^/cm^2^ and 3 × 10^4^/cm^2^, respectively. 2D hAMSC-CM, γ-hAMSC-CM and 3D hAMSC-CM were applied during reperfusion in a ratio 1:1 with normal medium specific for each cell type.

### 2.8. Intracellular ATP Measurement, ROS Detection and Apoptosis Assay

Intracellular ATP measurement was performed to detect the effects of cold ischemia and for cell viability analysis. For cold ischemia experiment, M1-like macrophages and THLE-2 cells were seeded in 96-well plates and incubated at 4 °C with 5% O_2_ exposition up to 24 h. Cells were harvested at each timepoint, analyzed and ATP levels have been measured by Cell Titer Glo 2.0 kit (Promega, Fitchburg, WI, USA) according to the manufacturer’s instructions. With the same protocol, cell viability was assessed after warm reperfusion of THLE-2 cells.

ROS production in reperfused M1-like macrophages has been assessed via ROS-Glo H_2_O_2_ Assay (Promega, USA) according to the manufacturer’s instructions.

Apoptosis of THLE-2 cells was measured with the ApoTox-Glo™ Triplex Kit (Promega, USA), which specifically detects caspase 3/7 activity by luminescence. Even in this case, the protocol has been performed in accordance with the kit’s instructions. Luminescence was measured with the Spark Plate Reader (Tecan, Zurich, Switzerland).

### 2.9. Analysis of Secreted Proteins

CM from reperfused cells was used for the detection of specific soluble factors, which have been detected by ELISA. In particular, ELISA assay was performed for the detection of IL-1β, TNFα, IL-10 and IL-13 (all kits from Merck, Darmstadt, Germany) according to the manufacturer’s protocols. Absorbance was assessed at 450 nm in the Spark Plate Reader (Tecan, Switzerland).

The levels of different cytokine and growth factors in each conditioned medium from primed hAMSCs (2D hAMSC-CM, 3D hAMSC-CM and γ-hAMSC-CM) were determined using Luminex™ magnetic bead technology with the ProcartaPlex Human Cytokine Chemokine Growth Factor Kit (Affymetrix, Santa Clara, CA, USA) according to the manufacturer’s instructions. Briefly, each analyte was quantified using a Luminex 200 instrument, which utilizes xMAP technology, multiple analyte profiling and xPONENT 4.2 software (Luminex Corp., Austin, TX, USA). The xMAP technology uses fluorescence-coded color magnetic microspheres coated with analyte-specific capture antibodies to simultaneously measure multiple analytes in a single specimen. Concentration of each factor was calculated from standard curves.

### 2.10. RNA Extraction and Gene Expression Analysis

Total RNA was extracted with the miRNeasy Mini Kit and treated with DNAse (Qiagen, Hilden, Germany) according to manufacturer’s instructions. The purity of isolated RNA was determined by OD260/280 using a Nanodrop ND-1000 (Thermo Fisher Scientific, Waltham, MA, USA). Subsequently, approximately 100 ng/µL of RNA was reverse-transcribed with the high capacity RNA-to-cDNA kit protocol (Applied Biosystems, Thermo Fisher Scientific, USA) by following manufacturer’s instructions.

We performed real-time PCR using cDNA as the template in a 20-μL reaction mixture containing TaqMan Universal Master Mix II (Thermo Fisher Scientific, USA), and specific primers were used as listed in Table 1. Expression of mRNA was quantified by PCR using StepOnePlus Real-Time PCR System (Applied Biosystems, Thermo Fisher Scientific, USA). GAPDH was used as a reference gene for the relative quantification, assessed by 2^−∆∆CT^ calculation for each mRNA.

### 2.11. Statistical Analysis

For the purposes of the current work, all values were represented as mean ± std. Statistical analysis has been performed using GraphPad Prism 9.0 (GraphPad Software, San Diego, CA, USA). In particular, one-way ANOVA test with multiple comparisons has been used according to the type of samples to compare. Statistical significance was considered at *p* < 0.05.

## 3. Results

### 3.1. Differentiation of Peripheral Blood Mononuclear Cells into M1-Like Macrophages and Establishment of an In Vitro Protocol of Ischemia/Reperfusion Injury

**Figure 1:**
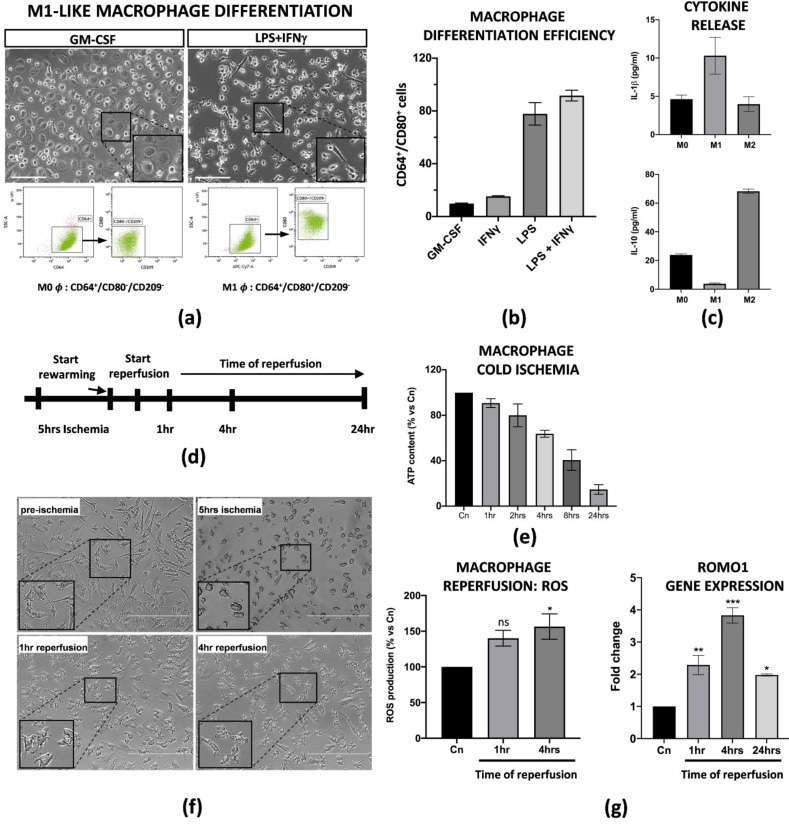
PBMC derived M1-like macrophages displayed an ischemic phenotype. (**a**) Flow cytometry analysis and image representation of CD14^+^ monocytes differentiated with GM-CSF alone or in combination with LPS + IFNγ (*n* = 3). Scale bar 75 μm. (**b**) Flow cytometry quantification of the M1-like differentiation efficiency upon treatment with GM-CSF, LPS or IFNγ alone, and LPS + IFNγ co-treatment. Data are represented as % of CD64^+^/CD80^+^ differentiated cells for each condition treatment (*n* = 3). (**c**) IL-1β and IL-10 protein quantification in the conditioned medium of M0, M1 and M2 macrophages. Protein concentration has been calculated according to the standard curve of each ELISA assay (*n* = 3). (**d**) Schematic representation of the IRI protocol applied to M1-like macrophages. (**e**) Intracellular ATP quantification in M1-like macrophages challenged with cold ischemia at different timepoints. Data are represented as % of ATP in the experimental conditions vs. control (Cn) not induced to ischemia (*n* = 4). (**f**) Image representation of M1-like macrophages subjected to full IRI protocol. Scale bar 200 μm. (**g**) Left panel, quantification of ROS production in M1-like macrophages subjected to full IRI. Data are represented as % of ROS production in experimental samples vs. unperturbed control cells. Statistical significance has been calculated via ordinary one-way ANOVA (Dunnet’s multiple comparisons test, *n* = 4, * = *p* < 0.05; ns = not significant). Right panel, gene expression analysis of Romo1 gene on reperfused M1-like macrophages at different timepoints. The comparison has been conducted by using the ∆∆CT method and normalized to GAPDH transcript. Statistical significance has been calculated via ordinary one-way ANOVA (Dunnet’s multiple comparisons test, *n* = 3, * = *p* < 0.05; ** = *p* < 0.005; *** = *p* < 0.0005).

Liver resident Kupffer cells with a pro-inflammatory M1 phenotype are the first ones to be perturbed during IRI in transplanted patients and in animal models [23,24]. Thus, in order to set up an in vitro model of IRI, we applied established protocols from Tarique et al. [22] to obtain M1-like macrophages from peripheral blood mononuclear cells (PBMCs). We obtained M1-like macrophages from CD14^+^ monocytes after treatment with GM-CSF (five days) and LPS + IFNγ (four subsequent days). In particular, a flow cytometry analysis demonstrated that LPS + IFNγ induced a 95% induction of M1-like macrophages CD64^+^/CD80^+^/CD209^−^ in comparison with the single cytokine treatments alone (Figure 1a,b). More importantly, we found that the differentiated M1-like macrophages were functionally active as they released high levels of pro-inflammatory IL-1β in the conditioned media, and, on the contrary, very low levels of IL-10, in comparison with macrophages with an M0 or M2 phenotype (Figure 1c). In order to define the ideal conditions for the IRI protocol, we first tested how differentiated M1-like macrophages reacted at cold ischemic conditions. Briefly, the cells were subjected to a preservation medium at 4 °C under 5% O_2_ exposition for a time range of 1 to 24 h. The intracellular ATP content was used to measure the ischemic effects at each timepoint. We found that the macrophages reduced their intracellular ATP level in a time-dependant manner, with a peak of 80% reduction at 24 h. For our purposes, we decided to perform 5 h of cold ischemia since the ATP reduction was about 50% (Figure 1e and Supplementary Figure S1c). After a brief rewarming, static reperfusion was performed in macrophage media at 37 °C and the effects of the physiological restoring conditions were measured at 1, 4 and 24 h of reperfusion (Figure 1d). Ischemia protocol strongly changed the morphology of the macrophages as they lost their typical appearance in the culture (Figure 1f). Consistent with the literature, warm reperfusion determined a 50% increased production of ROS at 4 h (Figure 1g, left panel), along with increased transcription of Romo1, the gene involved in ROS production (Figure 1g, right panel).

**Figure 2:**
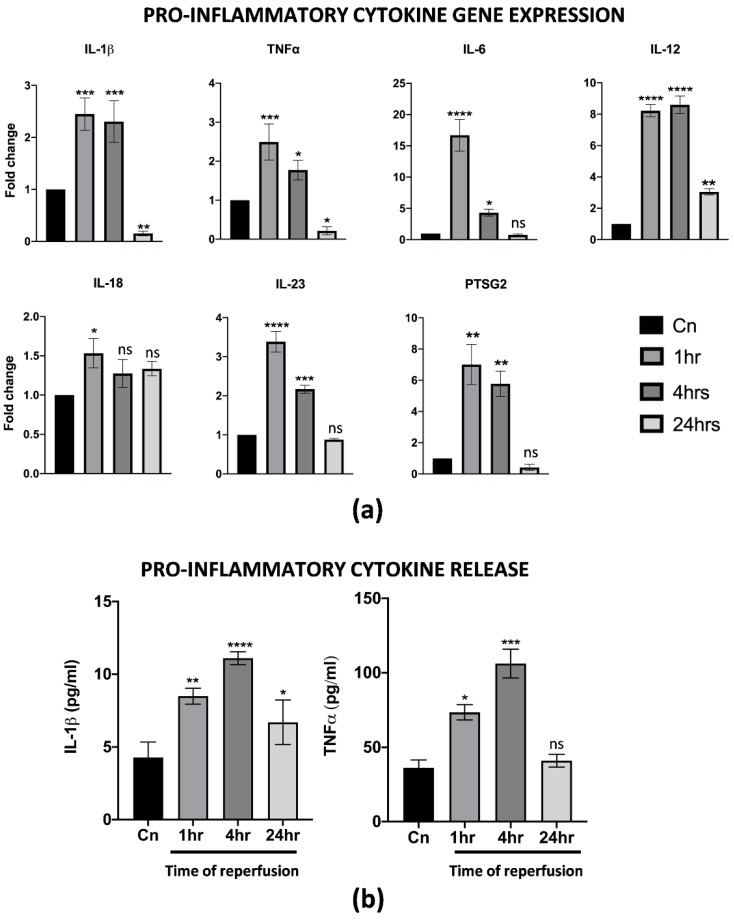
PBMC derived M1-like macrophages displayed a pro-inflammatory phenotype upon IRI. (**a**) Gene expression analysis of pro-inflammatory genes in reperfused M1-like macrophages at different timepoints. The comparison has been conducted by using the ∆∆CT method and normalized to GAPDH transcript. Statistical significance has been calculated via ordinary one-way ANOVA (Dunnet’s multiple comparisons test, *n* = 3, * = *p* < 0.05; ** = *p* < 0.005; *** = *p* < 0.0005; **** = *p* < 0.0001; ns = not significant). (**b**) IL-1β and TNFα protein quantification in the conditioned medium of reperfused macrophages at different timepoints. Protein concentration has been calculated according to the standard curve of each ELISA assay (*n* = 3). Statistical significance has been calculated via ordinary one-way ANOVA (Dunnet’s multiple comparisons test, *n* = 3, * = *p* < 0.05; ** = *p* < 0.005; *** = *p* < 0.0005; **** = *p* < 0.0001; ns = not significant).

One of the first events upon reperfusion is the activation of an inflammatory cascade, usually mediated, at least at first, by M1 macrophages resident in the liver. Thus, the effects of warm reperfusion were also assessed by a gene expression analysis and ELISA of specific inflammation markers. Interestingly, we found the up-regulation of several pro-inflammatory cytokines, including IL-1β, TNFα, IL-6, IL-12, IL-23 and PTSG2. In particular, the resulting increase in their transcription at 1 and 4 h of warm reperfusion, with a strong reduction at 24 h, was almost comparable to the non-reperfused control cells. To note, IL-18 changed in its transcription levels only at 1 h, while no differences were found throughout the other timepoints analyzed (Figure 2a). The ELISA assays on the CM from reperfused cells demonstrated that the IL-1β and TNFα release were strongly correlated with the gene expression assay (Figure 2b). All together, these data suggest that differentiated M1-like macrophages activated in vitro all the cellular pathways typically associated with IRI.

### 3.2. Establishment of In Vitro IRI Protocol on Hepatic Cells

**Figure 3:**
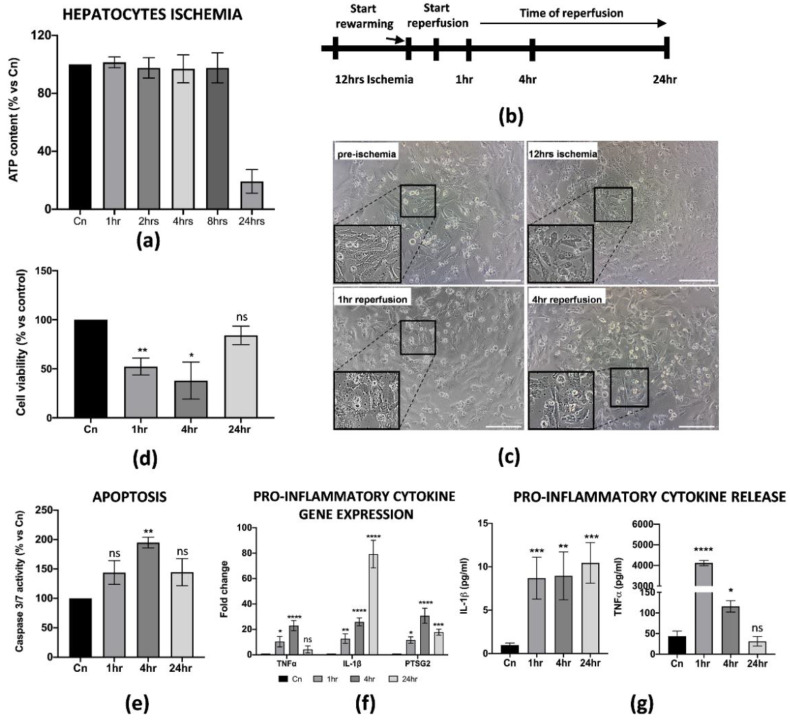
In vitro IRI on THLE-2 hepatocytes induced inflammation and apoptosis. (**a**) Intracellular ATP quantification in THLE-2 subjected to cold ischemia at different timepoints. Data are represented as % of ATP in the experimental conditions vs. control (Cn) not induced to ischemia (*n* = 4). (**b**) Schematic representation of the IRI protocol applied to THLE-2 hepatocytes. (**c**) Image representation of M1-like macrophages challenged with full IRI protocol. Scale bar 75 μm. (**d**) Cell viability analysis of THLE-2 reperfused at different timepoints. Data are represented as % vs. control (mean ± std). Statistical significance has been calculated via ordinary one-way ANOVA (Dunnet’s multiple comparisons test, *n* = 3, * = *p* < 0.05; ** = *p* < 0.005; ns = not significant). (**e**) Caspase 3/7 analysis in THLE-2 hepatocytes after reperfusion at the timepoints indicated. Data are represented % vs. control (mean ± std). Statistical significance has been calculated via ordinary one-way ANOVA (Dunnet’s multiple comparisons test, *n* = 3, ** = *p* < 0.005; ns = not significant). (**f**) Gene expression analysis of pro-inflammatory genes in reperfused THLE-2 hepatocytes at different timepoints. The comparison has been conducted by using the ∆∆CT method and normalized to GAPDH transcript. Statistical significance has been calculated via ordinary one-way ANOVA (Dunnet’s multiple comparisons test, *n* = 3, * = *p* < 0.05; *** = *p* < 0.0005; **** = *p* < 0.0001; ns = not significant). (**g**) IL-1β and TNFα protein quantification in the conditioned medium of reperfused THLE-2 hepatocytes at different timepoints. Protein concentration has been calculated according to the standard curve of each ELISA assay (*n* = 3). Statistical significance has been calculated via ordinary one-way ANOVA (Dunnet’s multiple comparisons test, *n* = 3, * = *p* < 0.05; ** = *p* < 0.005; *** = *p* < 0.0005; **** = *p* < 0.0001; ns = not significant).

During liver IRI, hepatic cell damage is due to several biological processes stimulated by the pro-inflammatory microenvironment generated at the early stages of reperfusion. The final result is the reduction in cell viability and apoptosis of the parenchyma, usually associated with the failure of a liver transplant. Thus, we thought it was necessary to establish an in vitro protocol that could better define the cellular and molecular behavior of hepatic cells upon reperfusion. For our purposes, we used the THLE-2 cell line, adult epithelial hepatocytes widely used for studies of liver biology and hypoxia/reoxygenation [25,26]. Cold ischemia experiments showed that THLE-2 cells were quite resistant to low temperatures and 5% O_2_ exposition as the intracellular ATP did not decrease if not at 12 h of cold ischemia (≅50% reduction) (Figure 3a and Supplementary Figure S1c). Thus, different from M1-like macrophages, in vitro IRI on hepatocytes has been performed with 12 h of cold ischemia in preservation media, followed by warm reperfusion at 1, 4 and 24 h checkpoints (Figure 3b). As expected, 12 h of cold ischemia altered the THLE-2 cell morphology (Figure 3c). These findings were further confirmed by the drastic reduction in cell viability at the early stages of reperfusion (Figure 3d), and by increased apoptosis at 4 h measured by caspase 3/7 activity (Figure 3e). In addition, we found that reperfused hepatocytes strongly up-regulated pro-inflammatory genes, including IL-1β, TNFα and PTSG2 (Figure 3f). Interestingly, the up-regulation pattern is comparable with the one for M1-macrophages, except for IL-1β as we did not observe any decrease at 24 h post-reperfusion. Finally, we confirmed by ELISA assays the release in the CM of pro-inflammatory IL-1β and TNFα proteins (Figure 3g). Taken together, these data suggest that we managed to generate an in vitro protocol of IRI on hepatic cells, thus showing cellular and molecular alterations strongly associated with the damage.

### 3.3. hAMSCs Pre-Conditioning Attenuates IRI on Differentiated M1-like Macrophages

**Figure 4:**
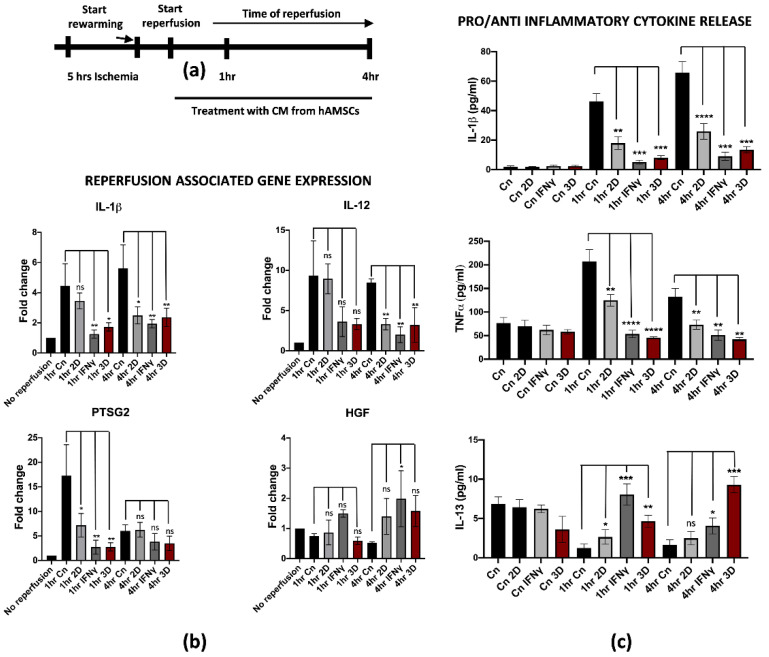
hAMSCs conditioned medium attenuates inflammation in reperfused M1-like macrophages. (**a**) Schematic representation of the modified IRI protocol applied to M1-like macrophages. (**b**) Gene expression analysis of pro-inflammatory genes in reperfused M1-like macrophages treated at different timepoints with pre-conditioned CM from hAMSCs. The comparison has been conducted by using the ∆∆CT method and normalized to GAPDH transcript. Statistical significance has been calculated via ordinary one-way ANOVA (Dunnet’s multiple comparisons test, *n* = 3, * = *p* < 0.05; ** = *p* < 0.005; ns = not significant). (**c**) IL-1β and TNFα and IL-13 protein quantification in the conditioned medium of reperfused M1-like macrophages treated at different timepoints with pre-conditioned CM from hAMSCs. Protein concentration has been calculated according to the standard curve of each ELISA assay (*n* = 3). Statistical significance has been calculated via ordinary one-way ANOVA (Dunnet’s multiple comparisons test, *n* = 3, * = *p* < 0.05; ** = *p* < 0.005; *** = *p* < 0.0005; **** = *p* < 0.0001; ns = not significant). The statistical analyses have been performed by comparing 1 h and 4 h Cn (black histograms) vs. the samples at the same timepoints treated with preconditioned CM-hAMSCs.

Recent studies pointed at the isolation and deep characterization of hAMSCs and their potential applications in regenerative medicine [27,28,29]. More importantly, we found that pre-conditioning hAMSCs by pre-treatment with IFNγ or by spontaneous 3D culture growth further stimulated the anti-inflammatory and regenerative properties, thus making hAMSCs an important biological tool for immunotherapies and tissue regeneration [21,30]. Based on this knowledge, we thought to evaluate the potential role of hAMSCs pre-conditioning in attenuating IRI damage on reperfused M1-like macrophages. For this purpose, previously isolated hAMSCs were cultured for 48 h with IFNγ or spontaneously grown in a 3D culture system. Pre-conditioned CM was collected and applied to M1-like macrophages during warm reperfusion at early timepoints (1 and 4 h of reperfusion, Figure 4a). We found that pre-conditioned CM down-regulated reperfusion-associated genes, including the pro-inflammatory IL-1β, IL-12 and PTSG2 (Figure 4b). Interestingly, the effects of the pre-conditioning were stronger when the hAMSCs were primed in comparison with CM derived from untreated hAMSCs (2D culture) both at 1 and 4 h of reperfusion. To note, no significant differences have been found between IFNγ and 3D pre-conditioning. Furthermore, the gene expression analysis showed an up-regulation of the HGF gene in M1-like macrophages reperfused with pre-conditioned media. In this case, the effect seemed to be more pronounced with IFNγ pre-conditioning at 1 h, while no differences were noticed at 4 h of reperfusion. This finding is in strong accordance with previous data from the literature, suggesting that HGF-met signaling activation in reperfused livers attenuates the damage from IRI [31]. Moreover, we tested whether the effect of pre-conditioning somehow affected the pro-inflammatory phenotype also at the protein level by measuring the cytokines concentration released in the CM. In accordance with the gene expression analysis, the IL-1β and TNFα protein release were significantly reduced after treatment with pre-conditioned CM in comparison with the control and 2D treatment (Figure 4c upper and middle panel). Interestingly, we found an overall increased release of IL-13 when M1-like macrophages were reperfused with pre-conditioned hAMSCs-derived CM compared to CM from 2D cultured hAMSCs. Interestingly, IL-13 is an anti-inflammatory cytokine that has been previously described to protect endothelial and hepatic cells from IRI [32,33] (Figure 4c, lower panel). Taken together, these data clearly demonstrate that hAMSCs pre-conditioning reverted the pro-inflammatory phenotype of M1-like reperfused macrophages.

### 3.4. hAMSCs Pre-Conditioning Improve Hepatocytes Cell Viability and Reduced Apoptosis of the Parenchyma during IRI

**Figure 5:**
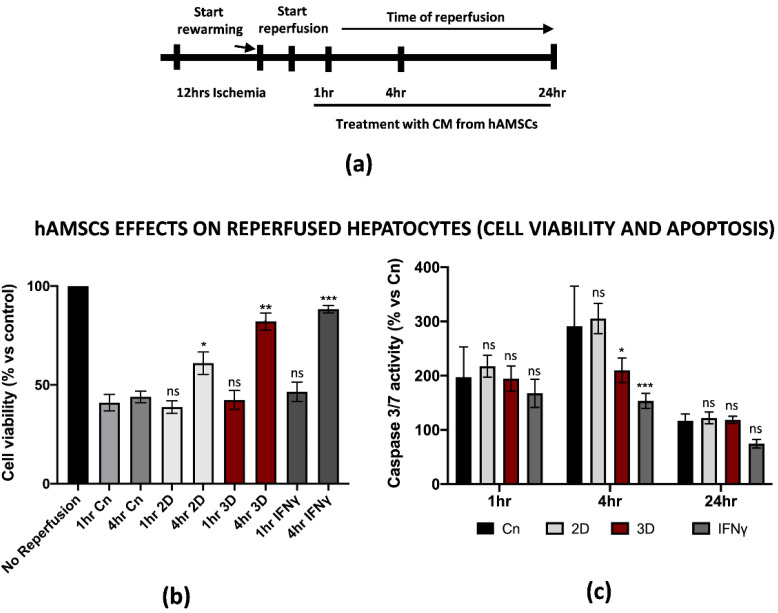
Conditioned medium from primed hAMSCs improved hepatic cell viability and reduced apoptosis after in vitro IRI. (**a**) Schematic representation of the modified IRI protocol applied to THLE-2 macrophages. (**b**) Cell viability analysis of THLE-2 reperfused at different timepoints with pre-conditioned CM from hAMSCs. Data are represented as % vs. control (mean ± std). Statistical significance has been calculated via ordinary one-way ANOVA (Dunnet’s multiple comparisons test, *n* = 3, * = *p* < 0.05; ** = *p* < 0.005; *** = *p* < 0.0005; ns = not significant). (**c**) Caspase 3/7 analysis in THLE-2 hepatocytes after reperfusion at the timepoints in presence of pre-conditioned CM from hAMSCs. Data are represented % vs. control (mean ± std). Statistical significance has been calculated via ordinary one-way ANOVA (Dunnet’s multiple comparisons test, *n* = 3, * = *p* < 0.05; *** = *p* < 0.0005; ns = not significant).

Along with the positive effects on the inflammation induced by M1-like macrophages during IRI, we asked whether hAMSCs pre-conditioning could attenuate the dramatic outcome of hepatic cells after warm reperfusion. For this reason, we decided to reperfuse THLE-2 cells in the presence of CM from IFNγ pre-conditioned or 3D cultured hAMSCs (1 to 24 h, Figure 5a). An intracellular ATP measurement assay showed that pre-conditioned CM improved the cell viability in hepatic cells reperfused with pre-conditioned CM. In particular, we found that 4 h of warm reperfusion increased the cell viability from 44% and 38% in the control and 2D treatment to 82% and 88% in the 3D and IFNγ pre-conditioning, respectively. To note, we have not found significant differences between the different pre-conditioning approaches after 1 h of reperfusion (Figure 5b). In addition, the same assay provided us another important piece of information as it suggested that 3D and IFNγ pre-conditioning might restore intracellular ATP levels. On the other hand, Caspase 3/7 activation assay demonstrated that pre-conditioned CM significantly reduced apoptosis at 4 h of warm reperfusion, while no differences have been found at 1 and 24 h (Figure 5c). However, the CM from 2D cultured hAMSCs did not impact the caspase activation. Of note, no significant differences were found when we compared the effects of 3D vs. IFNγ pre-conditioning. All together, these findings show how pre-conditioned CM from hAMSCs attenuated the effects of IRI in parenchymal hepatic cells.

### 3.5. Conditioned Medium Derived from Both 3D hAMSC and γ-hAMSC Cultures Showed Increased Production of Both Growth and Immunomodulatory Factors

**Figure 6:**
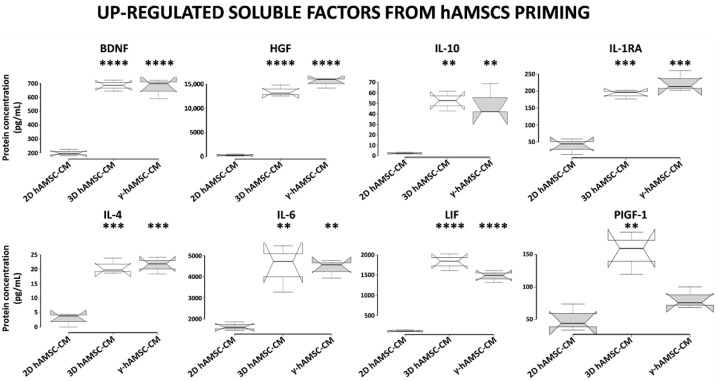
Secretion of functional factors in conditioned medium (CM) derived from two-dimensional (2D hAMSC-CM), three-dimensional (3D hAMSC-CM) and IFN-γ-treated (γ-hAMSC-CM) hAMSC cultures. The CM was collected after 48 h of culture. The concentrations of each factor were determined by multiplex-microbead immunoassay. Box plots of three independent experiments are displayed, where the horizontal bar represents the median, the box represents the interquartile range (IQR) and the whiskers represent the maximum and minimum values. Comparisons made by Dunnet’s *t*-tests. ** = *p* < 0.005; *** = *p* < 0.0005; **** = *p* < 0.0001 compared with 2D hAMSC-CM.

To investigate whether the different priming culture systems modified the composition of bioactive factors, we used a multiplex-microbead immunoassay to analyze the secretion of some proteins in the CM produced by hAMSCs grown in both 2D (with or without 200 IU/mL of IFNγ) and 3D cultures. In particular, we assessed the production of specific growth factors and cytokines, including BDNF, HGF, IL-10, IL-1RA, IL-4, IL-6, LIF and PIGF-1. As shown in Figure 6, when compared with hAMSCs 2D cultures, all the analyzed proteins were significantly up-regulated in both 3D hAMSC-CM and γ-hAMSC-CM except for PIGF-1 in γ-hAMSC-CM. More specifically, the concentrations of seven proteins, BDNF (3.4- and 3.3-fold), HGF (65.8- and 75.1-fold), IL-10 (18.6- and 18.1-fold), IL-1RA (3.7- and 4.3-fold), IL-4 (7.5- and 7.8-fold), IL-6 (2.7- and 2.6-fold) and LIF (15- and 12.1-fold), were significantly higher in both 3D hAMSC-CM and γ-hAMSC-CM, respectively, but there was no significant difference in proteins between 3D hAMSC-CM and γ-hAMSC-CM. Moreover, PIGF-1 was up-regulated 2.9-fold in 3D hAMSC-CM compared to 2D hAMSC-CM (Figure 6). These findings suggest that hAMSCs pre-conditioning clearly modified the release of bioactive factors that might be responsible for the outcome observed in the in vitro IRI model.

## 4. Discussion

In the last few years, the indicators of needing a liver transplant have been extended to all types of end-stage liver disease [34], thus increasing the number of potential recipients. However, the average waiting time for a liver transplant up to now is around 700 days [35], which means that (1) there is lack of organ availability, and (2) patients could develop some other disease that forces them to be removed from the list in the almost-two-year wait. The organ shortage has been overcome by extending the criteria of liver eligibility for transplantation, including steatotic or non-heart-beating organs; however, these features make them more susceptible to IRI. Thus, different approaches have been tried to reduce IRI effects in liver transplantation, including therapeutic strategies to reduce ROS effects, cytokine production and immune system activation. Unfortunately, despite the promising preliminary results, none of these attempts achieved the clinical effectiveness [36,37,38,39]. Because of the immunomodulatory and regenerative properties, hMSC therapy for liver IRI is currently under investigation as a valid approach to revert hepatic damages during organ transplantation [17,40]. However, although the major sources of hMSCs used for IRI studies are represented by bone marrow, adipose tissue and umbilical cord, there are no studies describing the role of amnion-derived MSCs (hAMSCs) in attenuating liver IRI. The hAMSCs derive from perinatal tissues, display the same properties of MSCs from other tissues [28,41] and might represent an important therapeutic tool considering the large number of cells or conditioned media that can be isolated or collected without any invasiveness [42].

For the purposes of our studies, we set up an in vitro protocol of ischemia/reperfusion on specific cell types, such as PBMC-derived M1-like macrophages and hepatic cells, with the final goal of testing the potential effect of hAMSCs in attenuating the IRI phenotype. In our in vitro model, the M1-like macrophages under cold ischemia and warm reperfusion displayed several molecular changes that are consistent with mitochondrial disfunction and a switch towards a pro-inflammatory phenotype that typically occurs in Kupffer cells during IRI. Among others, we found different pro-inflammatory and ROS-associated genes transcriptionally up-regulated in the reperfused cells, including IL-1β, TNFα, IL-6, IL-12, IL-23, PTSG2 and Romo1. Our findings suggested that PBMC-derived M1-like macrophages are a suitable model to study IRI in vitro despite the different genetic background of the donors. Interestingly, hAMSCs treatment during reperfusion did not markedly affect macrophages’ inflammation (2D columns in Figure 4). However, hAMSCs priming with the IFNγ or 3D culture condition strongly reduced the expression of IL-1β, IL-12 and PTSG2. Notably, the HGF up-regulation in the same samples is consistent with previous findings showing the HGF-Met signaling axis as protective towards liver IRI [31]. In addition, the ELISA analysis on the 3D hAMSC-CM and γ-hAMSC-CM from reperfused macrophages showed an increased release of IL-13 after reperfusion. This finding is in accordance with previous studies showing IL-13’s role in the protection from liver IRI [32,33], indicating that 3D hAMSC-CM and γ-hAMSC-CM treatment might revert macrophage polarization from a pro-inflammatory M1 state to an anti-inflammatory M2 one [43]. Our findings and hypothesis go in the same direction of the current literature on this topic as the identification of bioactive factors and molecules (proteins or miRNAs) able to stimulate M2 polarization is currently under investigation for new therapeutic strategies to improve liver IRI [44,45]. However, further experiments, beyond the purposes of this study, are required to address this question.

Along with an inflammatory phenotype, the final outcome of liver IRI, which is also the major cause of organ rejection, is represented by the apoptosis of parenchymal hepatic cells. Hepatic apoptosis might occur during cold ischemia, mainly due to ATP depletion; however, it continues at early reperfusion, thus inducing the release of damaged associated molecular patterns (DAMPs), which contribute to the activation of the pro-inflammatory cascade during IRI [46]. The final outcome of this biological process is the further apoptosis of endothelial and hepatic cells. Thus, the identification of factors that might arrest hepatic apoptosis is another crucial step in the reversion of the liver IRI phenotype. Our findings suggest that 3D hAMSC-CM and γ-hAMSC-CM not only restored the intracellular ATP levels but also showed a strong reduction in caspase 3/7 activity, thus improving the cell viability and reducing cell death. In a similar manner, Zheng and co-workers recently demonstrated that MSCs reverted hepatocellular apoptosis via the up-regulation of PINK1-dependent mitophagy [47].

Recently, MSCs pre-conditioning has been considered an important tool to improve MSCs therapeutic role towards inflammation and organ regeneration. Different studies have clearly demonstrated that different methods of pre-conditioning are related to the type of cell population or biological process that need to be targeted [30,48,49,50]. For instance, our group recently demonstrated via transcriptome analysis that hAMSCs pre-conditioning modulates an extensive number of genes associated with regenerative processes and signaling pathways [51]. Thus, the identification of bioactive factors in the pre-conditioned CM from MSCs is crucial for the definition of strategies to stimulate tissue repair. In our studies, 3D hAMSC-CM and γ-hAMSC-CM did not differ in terms of biological effectiveness: they both behaved similarly in reducing the inflammation and apoptosis of reperfused M1-like macrophages and hepatic cells. A luminex analysis of 3D hAMSC-CM and γ-hAMSC-CM identified few candidate factors that were up-regulated in both conditions when compared to the un-pre-conditioned cells. The list of factors includes, among others, BDNF, HGF, IL-10, IL-1RA, IL-4 and LIF. Interestingly, all of them have been shown to have a protective role towards IRI damage of different organs, including the liver, brain, heart and kidney [52,53,54,55,56,57]. These findings point to the possibility of dissecting the precise molecular mechanisms that the different bioactive factors might activate to exert their function in liver IRI.

## 5. Conclusions

Firstly, we established a novel method to induce IRI on human derived macrophages and hepatic cells. Then, we showed that hAMSCs pre-conditioning is a valid approach to reduce in vitro IRI damage in M1-like macrophages and THLE-2 hepatic cells. We have dissected the molecular changes occurring in specific cell types in vitro upon reperfusion with 3D hAMSC-CM and γ-hAMSC-CM and picked up a number of potential cytokines involved in the process of protection from IRI. Further investigations are necessary to dissect: (1) the precise molecular mechanisms regulating hAMSCs protection towards liver IRI, and (2) whether hAMSCs pre-conditioning might revert the IRI phenotype in liver transplantation. In this regard, extracellular vesicles, such as exosomes, are believed to be a crucial functional component of hMSC-derived CM, with a therapeutic role in several IRI models [58]. Moreover, MSC priming has been shown to enhance the therapeutic effects of extracellular vesicles [59]. Therefore, further studies and protocols need to be established to better define the therapeutic contribution that hAMSCs could give for the improvement of liver transplantation. Even if we are clearly aware that the major limitation of our in vitro models is the lack of cross-talk between the cell types involved in the IRI phenotype, these data support the idea to use hAMSCs to tackle liver transplantation rejection. The establishment of a more complex ex vivo “liver” organ, where cell–cell crosstalk can be assessed, will further improve our knowledge on how hAMSCs can affect cell behavior and interaction with the microenvironment.

## Figures and Tables

**Table 1:** List of TaqMan gene expression assays used for the study.

Assay ID	Gene Symbol	Description
#Hs01555410_m1	IL-1β	TaqMan^®^ Gene Expression Assay
#Hs00174128_m1	TNFα	TaqMan^®^ Gene Expression Assay
#Hs00174131_m1	IL-6	TaqMan^®^ Gene Expression Assay
#Hs01073447_m1	IL-12a	TaqMan^®^ Gene Expression Assay
#Hs01038788_m1	IL-18	TaqMan^®^ Gene Expression Assay
#Hs00372324_m1	IL-23a	TaqMan^®^ Gene Expression Assay
#Hs00603977_m1	Romo1	TaqMan^®^ Gene Expression Assay
#Hs00300159_m1	HGF	TaqMan^®^ Gene Expression Assay
#Hs00153133_m1	PTSG2	TaqMan^®^ Gene Expression Assay
#Hs02786624_g1	GAPDH	TaqMan^®^ Gene Expression Assay

## Data Availability

The datasets used and analyzed are available from the corresponding author on reasonable request.

## Supplementary Materials

The following supporting information can be downloaded at: https://www.mdpi.com/article/10.3390/cells11040709/s1, Figure S1: Isotype controls for Flow cytometry analysis (a); Flow cytometry plot of M2-like macrophages (b); ATP content analysis at 5hrs (macrophages) and 12hrs (hepatocytes) of cold ischemia (c).
