# Supplementary material for: Human Amnion-Derived Mesenchymal Stromal/Stem Cells Pre-Conditioning Inhibits Inflammation and Apoptosis of Immune and Parenchymal Cells in an In Vitro Model of Liver Ischemia/Reperfusion
Source: Cells. 2022 Feb 17;11(4):709. doi: 10.3390/cells11040709 (PMC8870407; doi:10.3390/cells11040709)
Supplement: Supplementary file 1 [file cells-11-00709-s001.zip › cells-1577036-supplementary.pdf]

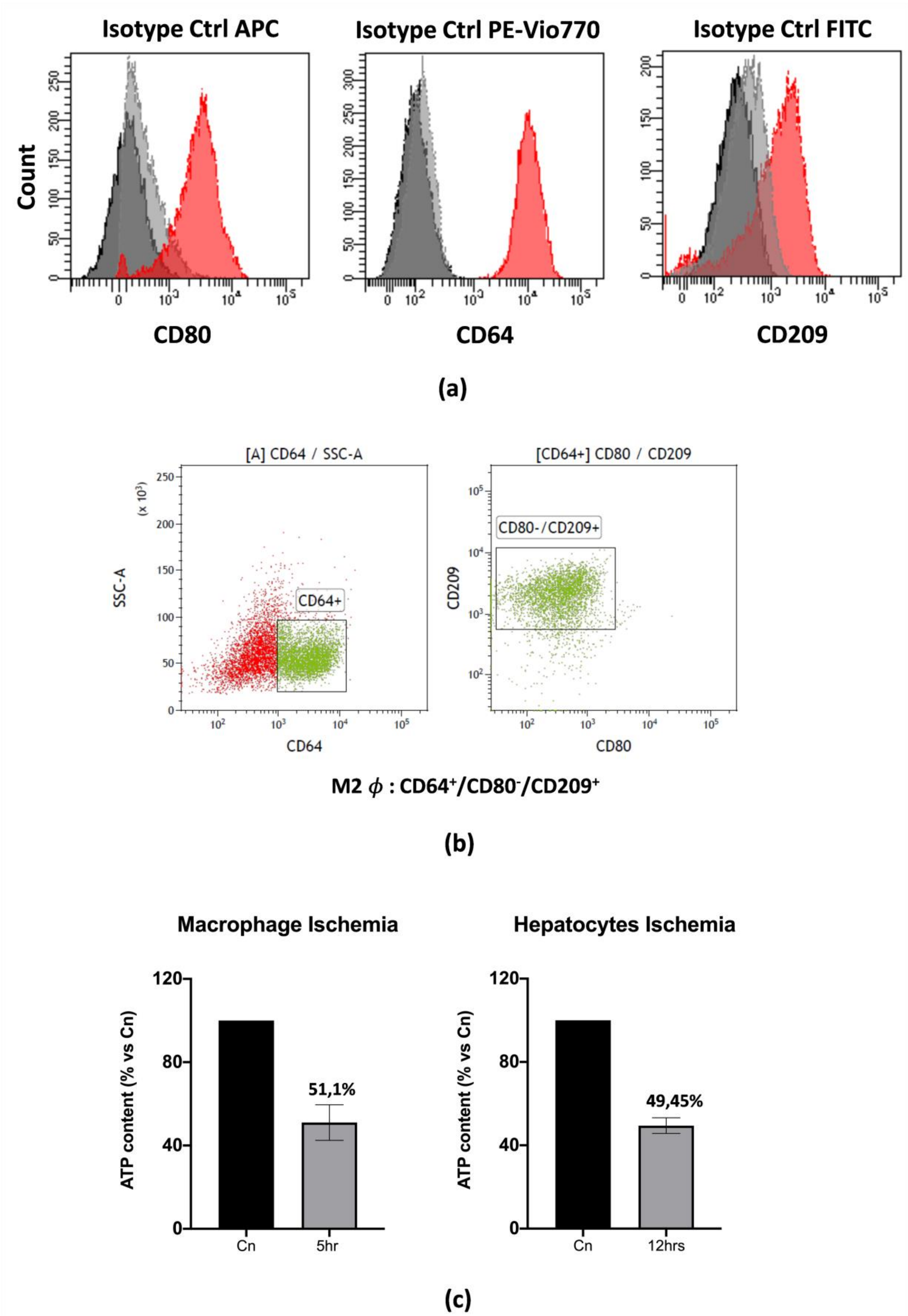

Figure S1: Isotype controls for Flow cytometry analysis **(a)**; Flow cytometry plot of M2-like macrophages **(b)**; ATP content analysis at 5hrs (macrophages) and 12hrs (hepatocytes) of cold ischemia **(c)**.
